# Color stability of CAD/CAM and additively manufactured resin materials: influence of surface treatment and layer thickness - an in vitro study

**DOI:** 10.1007/s44445-026-00217-0

**Published:** 2026-07-31

**Authors:** Tina Willmen, Merle Lankenau, Meike Stiesch, Philipp-Cornelius Pott

**Affiliations:** 1 Department of Prosthetic Dentistry and Biomedical Materials Research, Hannover Medical School, Hannover, Germany; 2 PRACTIS Clinician Scientist Program, Dean’s Office for Academic Career Development, Medizinische Hochschule Hannover, Hanover, Germany

**Keywords:** CAD/CAM, 3D printing, Color stability, Surface treatment, Composite resin

## Abstract

The increasing establishment of digital workflows in dentistry has brought both subtractive CAD/CAM processes and additive manufacturing techniques for fixed dental restorations into focus. This in vitro study aimed to compare the color stability of a CAD/CAM nanohybrid composite with a ceramic-filled, additively manufactured resin and to investigate how surface treatment and layer thickness influence this stability. Each thirty rectangular specimens in two different thicknesses (0.5 and 1.0 mm) were fabricated from two materials: a CAD/CAM nanohybrid composite (Grandio blocs) and a ceramic-filled 3D-printing resin (Permanent Crown Resin). Specimens of each material were assigned to three surface conditions: untreated, polished, or glazed. To simulate clinically relevant discoloration, specimens were stored for 28 days with one half immersed in coffee. Spectrophotometric measurements were performed daily during the first week and on days 14, 21, and 28. Changes in color (ΔE) were calculated. One-way ANOVA followed by Tukey’s post hoc test was performed. All experimental groups showed a time-dependent increase in color change relative to baseline during coffee storage, with the greatest increase occurring during the initial days. Within the limitations of this in vitro study, the CAD/CAM nanohybrid composite showed more favorable color stability than the additively manufactured resin. Surface treatment generally improved color stability, with more consistent effects in the additively manufactured material than in the CAD/CAM composite. In addition, specimens with reduced layer thickness showed greater color changes than thicker specimens.

**Graphical abstract:**

**Figure:**
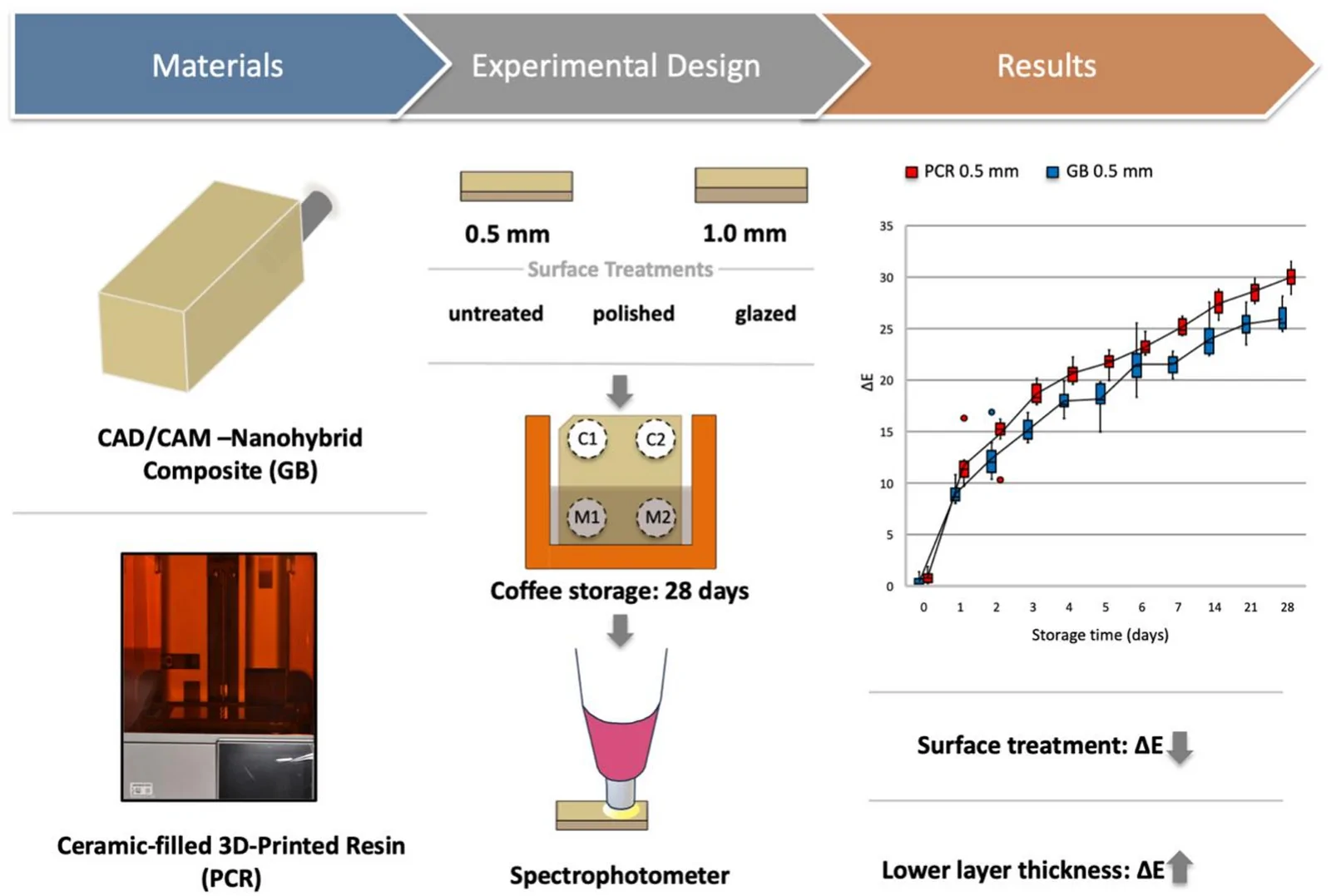

**Supplementary Information:**

The online version contains supplementary material available at https://doi.org/10.1007/s44445-026-00217-0.

## Introduction

The subtractive fabrication of dental restorations using CAD/CAM technology is already established in dental practice. 3D printing as an additive manufacturing process is also gaining increasing importance in recent years. A study of the American Dental Association highlights this development, showing that, in 2023, already 17% of dental practices in the USA were equipped with a 3D printer (Revilla-León et al. 2023).

The advent of digital workflows has supported the development of resin-based materials for fixed dental restorations, including CAD/CAM composites and additively manufactured resins intended for extended intraoral use. Compared to ceramics, the sintering process is omitted for composites, and the associated time savings as well as the reduced milling tool wear in the CAD/CAM process make composites economically attractive (Rauch and Gold 2018; Ruse and Sadoun 2014). The additive manufacturing process by means of 3D printing offers several further advantages compared to the subtractive CAD/CAM process: less waste is generated in the form of unprocessed material or worn milling tools (Della Bona et al. 2021). In addition, it shows higher cost efficiency regarding hardware investment and overall production (Van Noort 2012; Stansbury and Idacavage 2016; Della Bona et al. 2021).

For the aesthetics of dental restorations, correct tooth color plays an essential role alongside anatomical factors such as tooth size, shape, and position. Ideally, resin-based materials intended for extended intraoral use, including long-term provisional restorations and materials indicated by manufacturers for permanent crown applications, should exhibit high color stability to support long-lasting aesthetic outcomes. However, several studies report measurable color changes of composite materials over time as a limiting factor (Ulvestad 1978; Kul et al. 2021; Chakravarthy and Clarence 2018; Paolone et al. 2023). Their relevance is typically evaluated using perceptibility and acceptability thresholds. In the CIELAB system, ΔE*ab* describes the color difference derived from changes in *L*, a*, and b* [ΔE*ab = ((ΔL*)² + (Δa*)² + (Δb*)²)¹^,^²]. For ΔE*ab, 50:50% perceptibility and acceptability thresholds of 1.2 and 2.7, respectively, have been reported (Paravina et al. 2015). The intensity of color changes is influenced multifactorial by intrinsic factors such as the material composition of the composite itself (Al-Shami et al. 2023; Paolone et al. 2023). *Shin et al.* reported lower color stability of 3D-printed crown and bridge materials than CAD/CAM block materials after immersion in staining solutions (Shin et al. 2020). However, extrinsic discoloration of resin-based restorative materials is multifactorial and may be influenced by water sorption and solubility, resin matrix composition, filler type and filler content, and surface roughness (Paolone et al. 2023; Hashemzade et al. 2025; Osiceanu et al. 2025). In additively manufactured resins, post-processing conditions, particularly post-curing, may additionally affect the degree of conversion and staining susceptibility (Aktug Karademir et al. 2025). Patient-specific habits, such as tobacco consumption, the intake of coffee, red wine, or strongly staining foods such as curry, have also been reported by several authors to influence color stability (Ulvestad 1978; Chakravarthy and Clarence 2018; Kul et al. 2021; Wasilewski et al. 2010). Various in vitro studies demonstrate that color stability depends on the type of staining exposure. The most pronounced color changes were measured with red wine, followed by coffee (Guler et al. 2005; Şişmanoğlu 2022). Although red wine has been reported to cause more pronounced discoloration than coffee, coffee was chosen as the staining medium because it is frequently consumed and widely used in in vitro studies assessing staining-induced color changes. *Şişmanoğlu et al.*. showed that mechanical brushing significantly reduced discoloration of the nano-filled composite resin and achieved color recovery within the acceptability threshold for coffee-stained specimens (Şişmanoğlu 2022). Another study by *Lauvahutanon et al.* demonstrated that the positive effect of polishing correlates with the composite material used; thus, color changes in CAD/CAM composites were in most cases only extrinsic and reversible (Lauvahutanon et al. 2017). *Sagsoz et al.* investigated the importance of surface polishing for the color stability of CAD/CAM composites. They compared the color stability of four CAD/CAM composites using different polishing procedures and without polishing and showed that material-specific polishing contributed decisively to color stability (Sagsoz et al. 2016). Nam et al. also showed that surface treatment of 3D-printing resins intended for long-term intraoral use in the form of glazing has a positive effect on color stability (Nam et al. 2024). In addition to material- and surface-related factors, layer thickness can influence how discoloration-related color changes become visible (Lee et al. 2022). Thin resin-based restorations generally allow more light to pass through and can therefore make discoloration appear more pronounced. Greater material thickness, on the other hand, can more effectively mask superficial or deeper discoloration. Since extrinsic pigments can penetrate resin-based materials to varying degrees depending on the material, layer thickness should be taken into account when interpreting color changes following exposure to staining agents.

The presented in vitro study compares the color stability of the CAD/CAM nanohybrid composite Grandio blocs (GB) (VOCO GmbH, Cuxhaven, Germany; Shade A2, LT [low translucency], block size 14 L) and the ceramic-filled resin intended for 3D printing, Permanent Crown Resin (PCR) (Formlabs Inc., Somerville, MA, USA; Shade A2), using coffee as a staining medium to simulate clinically relevant discoloration. The following hypotheses were tested: (1) Color stability of GB is higher than that of PCR. (2) Surface treatment in the form of polishing or glazing leads to improved color stability of both materials. (3) Increased layer thickness results in improved color stability of both materials.

## Materials and methods

The study was conducted at the Department of Prosthetic Dentistry and Biomedical Materials Science, Hannover Medical School, Hannover, Germany.

### Specimen fabrication

**Fig. 1:**
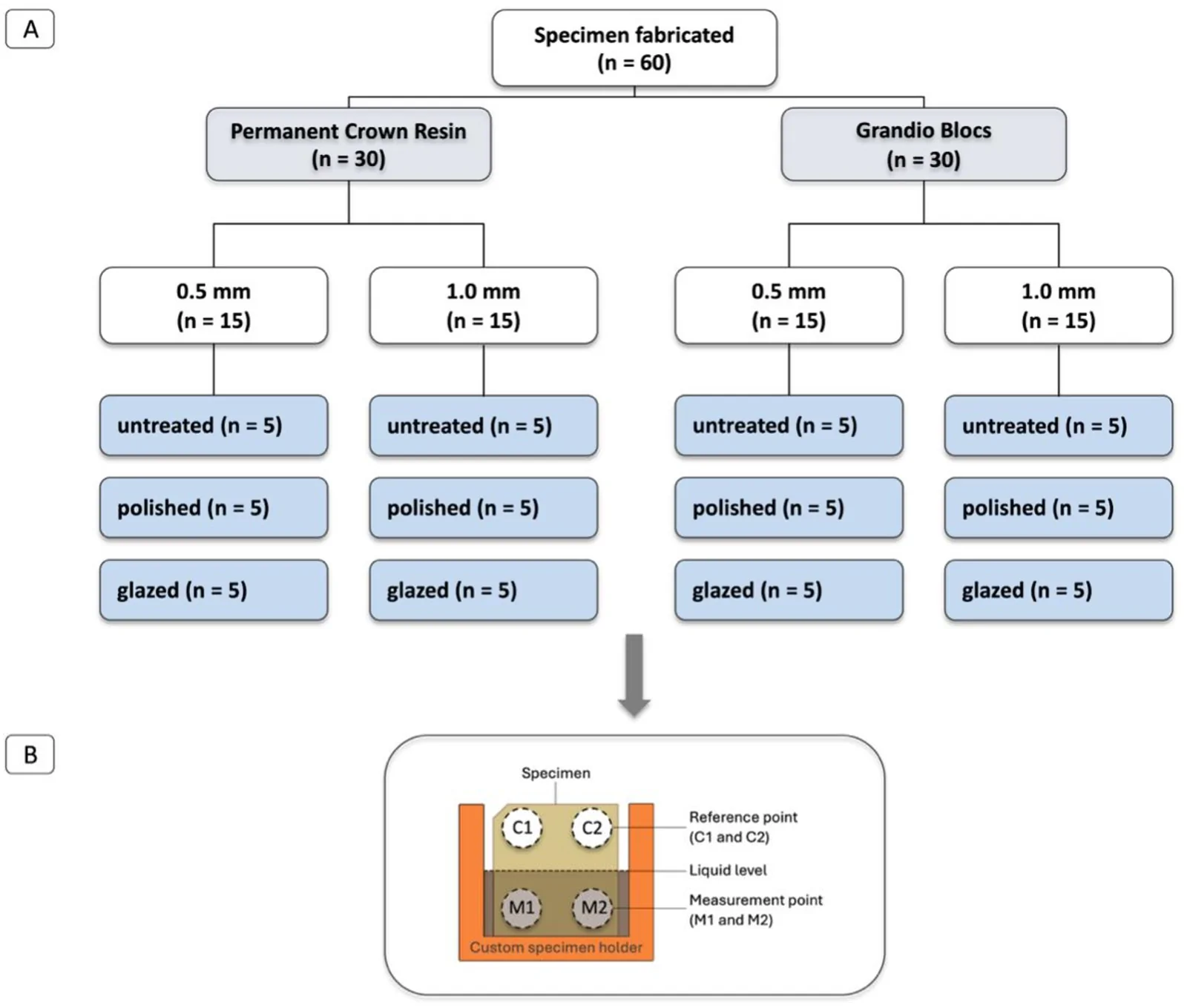
Experimental design. (**A**) A total of 60 specimens were fabricated and allocated according to material (GB, PCR), layer thickness (0.5–1.0 mm), and surface treatment (untreated, polished, glazed), resulting in twelve experimental groups (n = 5 specimens per group). (**B**) For each specimen, two measurement points (M1, M2) were defined in the immersed area exposed to the staining medium, and two control points (C1, C2) were defined in the non-immersed area above the liquid level for color assessment

In the present in vitro study, a factorial experimental design including two restorative materials, two layer thicknesses, and three surface treatments was applied. The investigated factors were material (GB, PCR), layer thickness (0.5 mm and 1.0 mm), and surface treatment (untreated, polished, glazed), resulting in twelve experimental groups (Fig. 1).

According to manufacturer’s information, GB is a nanoceramic hybrid composite consisting of a highly filled polymer matrix with 86% inorganic fillers, which was specifically developed for CAD/CAM fabrication of permanent single-tooth restorations. PCR is a ceramic-filled, tooth-colored resin for 3D printing that is indicated by the manufacturer for the fabrication of permanent single crowns.

A total of 30 rectangular specimens were cut from GB 14 L CAD/CAM blocks using a precision cutting machine with a diamond saw Brilliant 220 (ATM Qness GmbH, Mammelzen, Germany). Due to the tapered geometry of the GB blocks, the lateral dimensions varied slightly within the block and ranged approximately from 14.5 × 14.5 mm to 14.8 × 14.8 mm. Fifteen GB specimens were prepared with a standardized layer thickness of 1.0 mm, and 15 specimens with a standardized layer thickness of 0.5 mm. Thirty PCR specimens were fabricated with designed lateral dimensions of 14.5 × 14.5 mm (+ 0.20/−0.00 mm) and specimen thicknesses of either 1.0 mm–0.5 mm using a Form 2 3D printer (Formlabs Inc., Somerville, MA, USA). The print layer thickness was 0.050 mm. Specimens were built on support structures in an oblique orientation rather than directly on the build platform. After printing, residual resin was removed by rinsing with isopropyl alcohol and water, followed by automated washing in 99% isopropyl alcohol for 3 min (Form Wash, Formlabs Inc., Somerville, MA, USA). The specimens were dried with oil-free compressed air and visually checked for residual uncured resin. Initial post-curing was carried out in a Form Cure unit (Formlabs Inc., Somerville, MA, USA) at 60 °C for 20 min under 405-nm light with the support structures still attached. The supports were subsequently removed using a rotating separating disc and a dental handpiece. As a white powdery residue remained on the specimen surfaces after the first post-curing cycle, the specimens were additionally air-abraded with glass beads at a maximum pressure of 1.5 bar. Afterwards, the specimens were placed flat in the same Form Cure and post-cured again for 20 min at 60 °C.

All specimens were flattened on one side by grinding with a 45-µm abrasive in a grinding/polishing device to obtain a standardized experimental baseline surface for subsequent surface treatment and color measurements. This step was considered a grinding procedure and was not intended to reproduce a clinically finished restoration surface. The opposite side was left in its original as-milled or as-printed condition and was not used for color measurements. For each material, the standardized ground baseline surface was assigned to one of three conditions: no further treatment, polishing, or glazing. In this study, “untreated” refers to specimens whose standardized ground baseline surface received no additional polishing or glazing. The polished specimens were treated in two steps using a polishing motor (Type W77 K, C. H. Wilh. Wassermann, Hamburg, Germany) with pumice paste (Steribim plus, BEGO Bremer Goldschlägerei Wilh. Herbst GmbH, Bremen, Germany) and polishing paste (Sheralux 715, SHERA, Lemförde, Germany). The polishing procedure was performed by a single operator using a standardized protocol. Each polishing step was carried out for approximately 30 s per specimen, with the polishing time controlled using a stopwatch. Manual pressure was kept as consistent as possible throughout the procedure. In addition, profilometric measurements were performed on randomly selected specimens to assess the reproducibility of the surface treatment. The glazing group was coated with Easy Glaze (VOCO GmbH, Cuxhaven, Germany). For GB, the glazing procedure was included as an experimental surface modification to enable a standardized comparison of surface treatment effects across both materials under identical in vitro conditions. However, this procedure is not specified as a manufacturer-recommended finishing protocol for GB and should therefore not be interpreted as a clinically recommended surface treatment for this material.

According to the manufacturer’s instructions, Easy Glaze was applied bubble-free to the slightly roughened and dried surface of the specimens using a brush. Subsequently, the glazed surfaces were cured for 30 s with a polymerization lamp (Bluephase G2, Ivoclar Vivadent, Schaan, Liechtenstein) placed with a minimal distance above the surface emitting visible light (> 500 mW/cm²). To ensure reproducible positioning during color measurements and standardized placement in the specimen holder, the upper left corner of each specimen was beveled at a 45° angle.

### Storage conditions

**Fig. 2:**
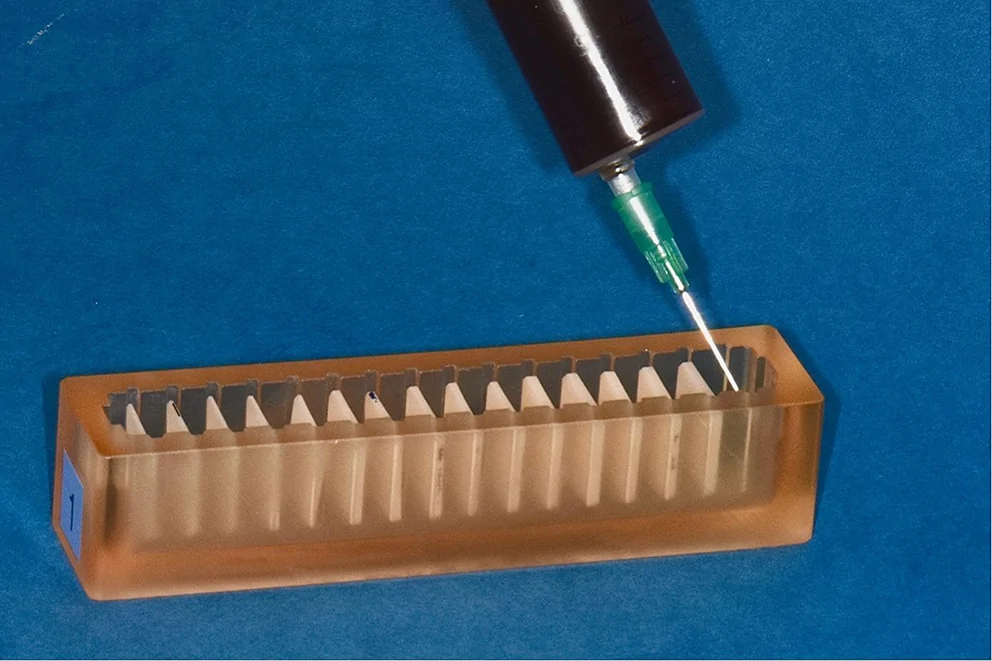
Custom-made specimen holder used for the storage of specimens during immersion in the staining solution. The image shows the positioning of the specimens within the holder while freshly brewed coffee is introduced using a syringe, allowing controlled immersion of the lower halves of the specimens

For storage of the specimens, special specimen holders were fabricated (Fig. 2). The specimens were placed in the holders and exposed for in total 28 days with their lower half to a staining solution of black coffee (Lungo 8 Intenso coffee capsules, Jacobs Douwe Egberts GmbH, Bremen, Germany). To ensure standardized preparation of the staining solution, the coffee was brewed using a capsule coffee machine (Nespresso system, Krups, Solingen, Germany) and Lungo 8 Intenso coffee capsules (Jacobs Douwe Egberts GmbH, Bremen, Germany). The freshly brewed coffee used as staining solution had a pH range of 5.40–5.45, measured using a pH meter 766 Calimatic (Knick Elektronische Messgeräte GmbH & Co. KG, Berlin, Germany), and a temperature of 50 ± 2 °C at the time of introduction into the specimen holders, measured using a digital thermometer Testo 935 (Testo SE & Co. KGaA, Titisee-Neustadt, Germany). The coffee was introduced using a syringe until the lower halves of the specimens were immersed. The staining solution was removed daily and replaced with freshly brewed hot coffee. During the experiment, the specimen holders were stored in a climate chamber (KBF 115, Binder, Tuttlingen, Germany) at a constant temperature of 37 °C and without light exposure to approximate intraoral conditions during the in vitro aging period. According to previously published data, approximately 24 h of continuous immersion in coffee have been proposed to roughly correspond to one month of average clinical coffee consumption (Peñate et al. 2021). Consequently, the 28-day immersion period may be regarded as an accelerated in vitro aging protocol approximating long-term coffee exposure, rather than as a direct clinical equivalent of two years of intraoral coffee consumption.

### Measurement of color values (L*, a*, b*)

Color measurements were performed using a spectrophotometer (VITA Easyshade V, VITA Zahnfabrik, Bad Säckingen, Germany). Before each measurement, the spectrophotometer was calibrated according to the manufacturer’s instructions. Prior to measurement, the specimens were carefully rinsed with water to remove loosely adherent residues of the staining solution and subsequently gently dabbed dry using absorbent paper towels. The surfaces were not wiped in order to avoid mechanical removal of stain deposits that could influence the color measurements.

Two predefined measurement points (M1, M2) within the area exposed to the staining solution were evaluated on each specimen. In addition to the immersed measuring points M1 and M2, corresponding non-immersed reference points (C1, C2) were also assessed on each specimen (Fig. 1). Measurements were conducted prior to immersion in the staining solution (baseline, day 0), followed by daily measurements during the first week (days 1–7). Additional measurements were performed after 14, 21, and 28 days, resulting in a total of eleven measurements for every measuring point. The color parameters L*, a*, and b* were recorded. At each measurement time point, the color values of the immersed measuring points were compared with those of the corresponding non-immersed reference points on the same specimen. ΔE*ab was calculated according to the following formula:

$$\:{\Delta}{E}_{\left\{ab\right\}}^{*}=\sqrt{\left\{{\left({\Delta}{L}^{*}\right)}^{2}+{\left({\Delta}{a}^{*}\right)}^{2}+{\left({\Delta}{b}^{*}\right)}^{2}\right\}}$$

The CIELAB color difference formula (ΔEab) was used to quantify color changes, as this parameter has been widely applied in previous studies on the color stability of resin-based dental materials and therefore allows direct comparison with earlier data. Moreover, the primary aim of the present study was to compare relative color changes between materials, layer thicknesses, and surface treatments under standardized in vitro conditions.

With five specimens per experimental group and two paired comparisons per specimen, ten ΔE values were calculated per group and time point. Across all experimental groups and time points, a total of 1320 ΔE values were calculated. All measurements were performed under controlled lighting conditions in a room without daylight using a daylight lamp (CL-LED256, Camlink/Nedis BV, Den Bosch, Netherlands). The measurements were carried out on a standardized white background.

### Statistical analysis

Statistical analysis was performed to evaluate differences in color change (ΔE) between the experimental groups. The independent variables were material (GB, PCR), layer thickness (0.5 mm and 1.0 mm), and surface treatment (untreated, polished, glazed), resulting in twelve experimental groups. The analysis aimed to assess changes in color over time within each experimental group and to compare the experimental groups with one another at predefined measurement time points. Baseline was defined as day 0, and days 1–7, 14, 21, and 28 were defined as follow-up time points. Within-group comparisons were performed between baseline and follow-up, between consecutive follow-up time points and across selected longer follow-up intervals to characterize the time course of color change. Between-group comparisons at corresponding time points were used to evaluate the effects of material, surface treatment, and layer thickness. To structure these analyses, measurement groups were defined. To support the sample-size rationale for the planned between-group comparisons, a theoretical estimate was performed using a one-way ANOVA model with twelve groups, a type I error level of 0.05, a target power of 0.80, and an expected large effect size according to Cohen (f = 0.40). Under these assumptions, approximately 116 observations would be required. Therefore, ten valid ΔE observations per group and a total of 120 observations were considered adequate for the primary between-group comparisons. Due to the loss of two specimens in subgroup GB-0.5 mm with glazed surface on day 3 and day 21, reduced number of measurements were possible at later follow-up time points in this group (Supplementary Table 1).

Normal distribution of the data and homogeneity of variances were assessed using the Kolmogorov–Smirnov test and Levene-Analysis. Differences in color change between measurement groups were analyzed using one-way analysis of variance (ANOVA). Pairwise comparisons were performed using Tukey’s post hoc test. A significance level of *p* < 0.05 was applied for all statistical tests. Results are presented as mean values with standard deviation. Statistical analyses were performed using SPSS Statistics for Windows, version 29.0.2.0 (IBM Corp., Armonk, NY, USA).

## Results

The color changes (ΔE) of the specimens were investigated for both materials as a function of surface treatment, layer thickness, and storage duration. In the glazed GB 0.5-mm subgroup, one specimen remained assessable up to day 2 but could not be included from day 3 onward because of chipping of the glaze layer during storage. A second specimen remained assessable up to day 14 but was no longer available from day 21 onward because of further glaze loss. Consequently, reduced numbers of ΔE values were available for this subgroup at later follow-up time points.

**Fig. 3:**
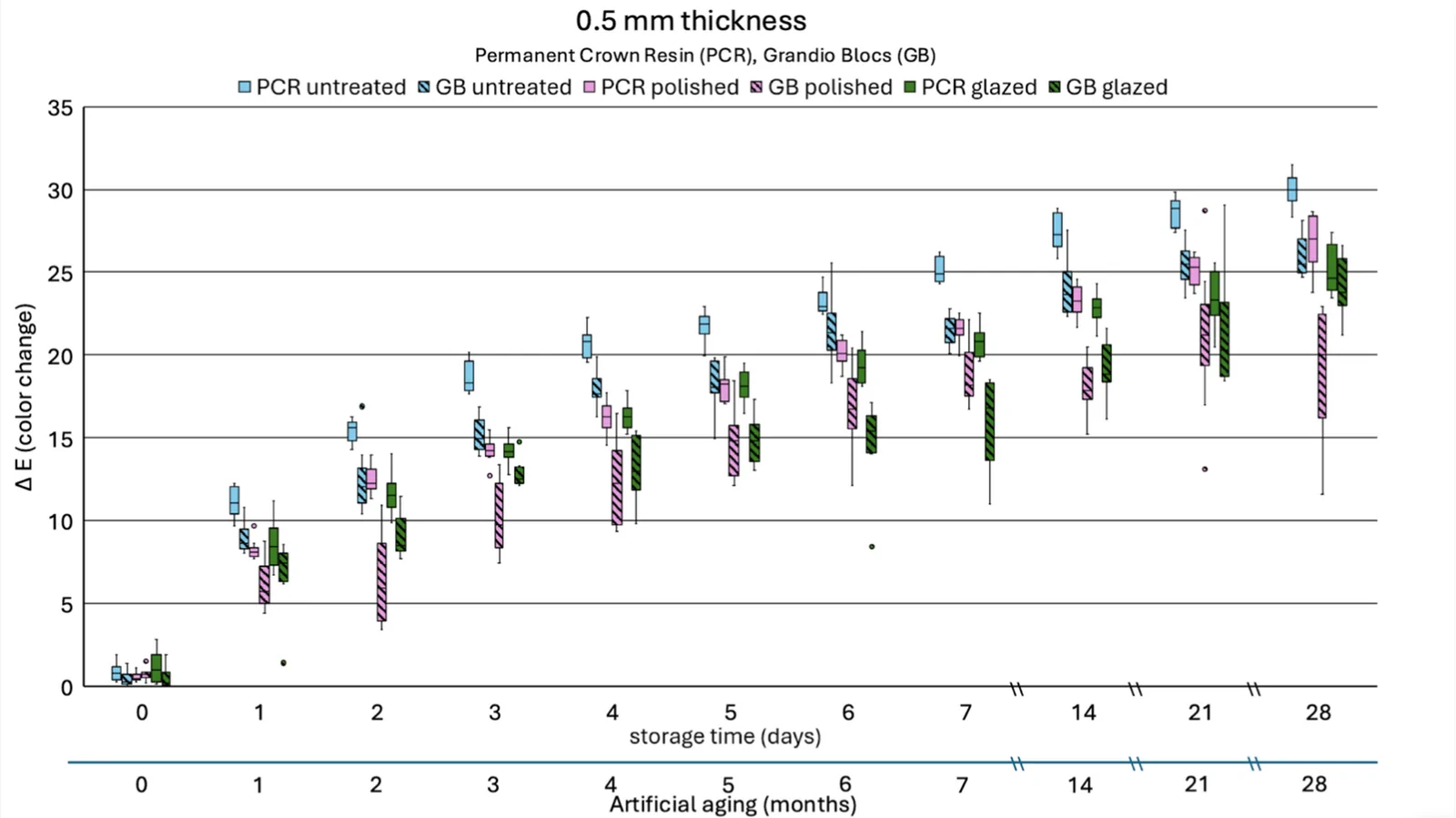
Color change (ΔE) over storage time for specimens with a layer thickness of 0.5 mm. Boxplots show the ΔE values of the investigated materials (PCR and GB) with different surface treatments (untreated, polished, glazed) at eleven measurement time points during storage in coffee

**Fig. 4:**
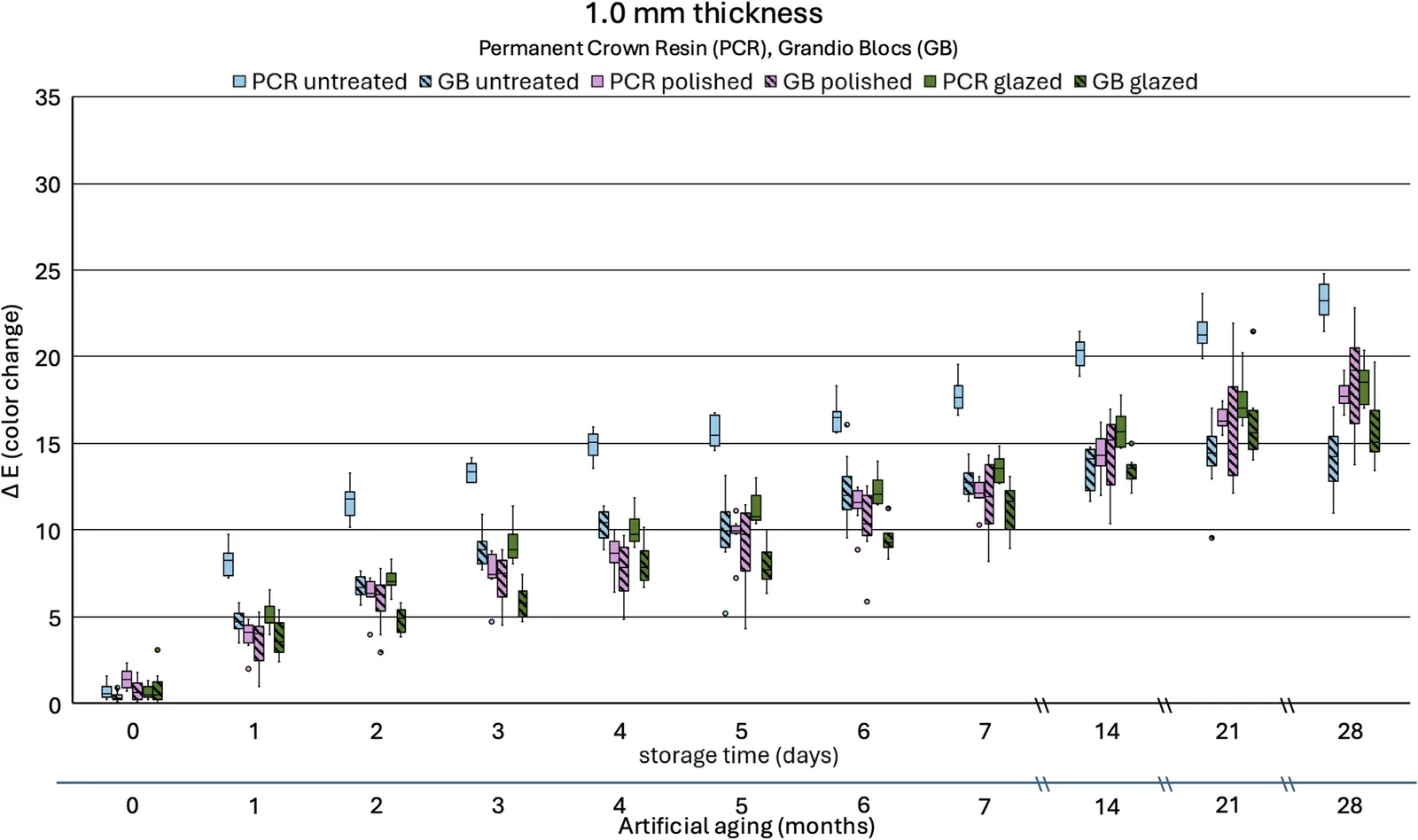
Color change (ΔE) over storage time for specimens with a layer thickness of 1.0 mm. Boxplots show the ΔE values of the investigated materials (PCR and GB) with different surface treatments (untreated, polished, glazed) at eleven measurement time points during storage in coffee

### Time-dependent color changes

After 24 h, the mean ΔE values exceeded the 50:50 clinical acceptability threshold of ΔE*ab = 2.7 in all experimental groups (Paravina et al. 2015). All groups except the polished 1.0-mm GB group also exceeded the commonly used threshold of ΔE*ab = 3.7 (Khashayar et al. 2014). Across all groups, mean ΔE values after 24 h ranged from 3.62 ± 1.31 to 11.10 ± 0.86. After 48 h, the acceptability threshold was exceeded in all experimental groups. Consistent with this pronounced early increase, 11 of the 12 groups showed a significant increase in ΔE between day 0 and day 1 (all *p* ≤ 0.032), whereas no significant difference was detected in this interval for the polished 1.0-mm PCR group (*p* = 0.238).

The most pronounced early discoloration dynamics were observed in the 0.5-mm groups, particularly in the PCR groups as well as in the untreated 0.5-mm GB group. Accordingly, between day 1 and day 2, a further significant increase was still detectable in five subgroups: the untreated 0.5-mm GB group, the untreated 1.0-mm PCR group, and all 0.5-mm PCR groups (all *p* ≤ 0.008), whereas the remaining seven subgroups showed no significant difference in this interval (all *p* ≥ 0.383). In most of these remaining subgroups, further significant increases became apparent only over the broader interval from day 1 to day 3 (all *p* < 0.001). The glazed 1.0-mm GB group represented the only exception, as neither between day 1 and day 2 (*p* > 0.999) nor between day 1 and day 3 (*p* = 0.767) was a significant difference observed; in this group, a further significant increase was first detected between day 1 and day 4 (*p* < 0.001).

Of the five subgroups that still showed a significant increase between day 1 and day 2, this remained significant between day 2 and day 3 only in the untreated 0.5-mm PCR group (*p* = 0.002). In the other four subgroups, the comparison between day 2 and day 3 was not significant (all *p* ≥ 0.085), whereas further significant increases again became detectable over the interval from day 2 to day 4 (all *p* ≤ 0.001). From day 3 to day 6, 11 of 12 subgroups showed a further significant increase (all *p* ≤ 0.007); the only exception was the glazed 0.5-mm GB group, for which no significant difference was observed in this interval (p **>** 0.999). Significant differences were still detectable between day 6 and day 14 in 9 of 12 subgroups (all *p* ≤ 0.024), whereas no significant changes were observed in this interval for the untreated 1.0-mm GB group, the untreated 0.5-mm GB group, and the polished 0.5-mm GB group (all *p* ≥ 0.311). In the later course, the number of significant differences decreased further. Between day 7 and day 14, a significant difference was observed only in the glazed 0.5-mm GB group (*p* = 0.019), whereas the remaining subgroups showed no significant differences in this interval (all *p* ≥ 0.233). Between day 14 and day 21, only the polished 0.5-mm GB group still showed a significant increase (*p* = 0.007), and between day 21 and day 28 only the polished 1.0-mm GB group (*p* = 0.018), whereas no significant differences were observed in any of the remaining subgroups (all *p* ≥ 0.797). Over the longer interval from day 14 to day 28, however, significant differences remained detectable in five subgroups: the polished 1.0-mm GB group, the glazed 0.5-mm GB group, the untreated 1.0-mm PCR group, and the polished 1.0-mm and 0.5-mm PCR groups (all *p* ≤ 0.019), whereas the remaining seven subgroups showed no significant changes during this period (all *p* ≥ 0.128). Overall, these findings indicate that changes in color did not follow a uniform linear day-to-day course but were characterized by a pronounced early increase followed by subgroup-specific slowing of the increase beginning at different time points.

### Group differences according to material, surface treatment, and layer thickness

Comparing the materials, at identical layer thickness and surface treatment, PCR specimens descriptively showed higher ΔE values than the corresponding GB specimens throughout the observation period. The material-related difference was most consistent in the untreated 1.0-mm groups, in which PCR exhibited significantly higher ΔE values than GB from day 1 onward (all *p* < 0.001), as well as in the polished 0.5-mm groups, which differed significantly from day 2 onward (all *p* ≤ 0.039). In contrast, no significant material-related difference was observed for the polished 1.0-mm groups at any time point after baseline (all *p* > 0.999). In the untreated 0.5-mm groups and in both glazed group pairs, PCR likewise showed higher ΔE values than GB, although these differences were not significant at every time point.

Surface treatment effects differed between materials. In PCR specimens, untreated surfaces consistently showed the highest ΔE values over time at both layer thicknesses. Relative to polished surfaces, untreated PCR specimens exhibited significantly higher ΔE values from day 1 onward (1.0 mm: all *p* < 0.001; 0.5 mm: all *p* ≤ 0.032). Relative to glazed surfaces, untreated PCR specimens likewise showed significantly higher ΔE values throughout the observation period after baseline in the 1.0-mm groups (all *p* ≤ 0.015) and from day 2 onward in the 0.5-mm groups (all *p* ≤ 0.001), whereas no significant difference was detected at day 1 (*p* = 0.191). No significant differences were observed between polished and glazed PCR specimens at any time point after baseline (1.0 mm: all *p* ≥ 0.998; 0.5 mm: all *p* ≥ 0.988).

In GB specimens, the surface-related effect was less consistent. In the 1.0-mm groups, significant differences were limited to day 3 between untreated and glazed specimens (*p* = 0.017) and to day 28 between untreated and polished specimens (*p* < 0.001), whereas all remaining comparisons were non-significant (all *p* ≥ 0.055). In the 0.5-mm groups, untreated specimens showed significantly higher ΔE values than polished specimens at all time points after baseline (all *p* ≤ 0.048) and glazed specimens at day 2 and from day 4 to day 21 (all *p* ≤ 0.009), while no significant differences were found at day 1, day 3, or day 28 (all *p* ≥ 0.722). Polished and glazed 0.5-mm GB specimens differed only at day 28, when glazed specimens showed higher ΔE values than polished specimens (*p* < 0.001).

In all experimental groups, samples with a layer thickness of 0.5 mm exhibited higher ΔE values than samples with a layer thickness of 1.0 mm. This difference was significant for all PCR groups as well as for the untreated and glazed GB groups at all time points after baseline (all *p* ≤ 0.020), whereas the polished GB groups did not differ significantly on Day 1, Day 2, and Day 28 (all *p* ≥ 0.316), as the comparison of Figs. 3 and 4 also illustrates.

## Discussion

The present study demonstrated material-, surface-, and thickness-dependent differences in color change during coffee storage.

### Methodological considerations of the experimental design

The presented study was designed as an in vitro investigation to evaluate the color stability of digitally manufactured restorative materials under standardized laboratory conditions. Coffee was selected as the staining medium because it is among the most frequently consumed beverages and its pronounced discoloration potential for composite-based dental materials is well documented. Previous studies have shown that the chromogenic components of coffee can lead to measurable color changes in resin-based restorations (Song et al. 2020; Bayindir et al. 2012; Taşın et al. 2022).

Continuous storage of the specimens in coffee was used as an accelerated aging model to simulate long-term exposure to staining beverages under standardized conditions. According to *Peñate et al.*., storage in coffee for 24 h approximately corresponds to one month of clinical coffee consumption (Peñate et al. 2021). The 28-day immersion period should be understood as a rough approximation of long-term exposure to coffee and not as a direct clinical time equivalent. This model enabled a standardized comparison of the materials and surface treatments under investigation.

Another methodological aspect concerns specimen drying prior to color measurement. In the present study, specimens were gently dabbed dry after rinsing in order to remove loosely adherent liquid residues while avoiding mechanical wiping of the surface. This procedure was chosen because the manufacturer recommends measurement on a dry surface. Nevertheless, it must be considered that the extent of residual surface moisture or adherent staining deposits may influence the recorded color values and therefore represents a method-inherent limitation. In addition, the dabbing process itself may have had a slight cleaning effect by removing loosely adhering discoloration from the surface. Although this effect was likely less pronounced than one would expect from wiping or brushing, it cannot be ruled out that the actual extent of the discoloration is thus slightly underestimated.

Surface treatment of the specimens also requires consideration from a methodological perspective. In the present study, only one side of each specimen was polished, whereas the opposite side remained untreated. This approach was chosen to approximate the clinical situation of restorative treatments, in which only the external surface is finished and polished, whereas the internal surface intended for cementation or adhesive bonding is generally not polished but conditioned for fixation. This procedure is an established method in experimental studies on the color stability of dental restorative materials and has been used in comparable studies (Yildirim and Recen 2021; Lauvahutanon et al. 2017).

In addition, two different material thicknesses were investigated in order to simulate clinically relevant restoration thicknesses. In clinical practice, restorations such as veneers or minimally invasive crowns may exhibit reduced material thicknesses. Thinner restorative layers may allow increased light transmission and may therefore enhance the visibility of discoloration processes.

### Material-related color stability

The first hypothesis was confirmed for the untreated, polished and glazed 0.5 mm groups, as well as the untreated and glazed 1.0 mm groups, since the PCR groups generally exhibited higher ΔE values than the corresponding GB groups. However, this was not the case for the polished 1.0-mm groups, for which no material-related difference was observed.

These findings are consistent with a recent systematic review and meta-analysis comparing additively and subtractively manufactured resin-based fixed dental restorations. The review reported lower color stability of additively manufactured resin-based materials after aging than of milled materials (Mosaddad et al. 2026). Similar observations have also been reported in individual in vitro studies demonstrating greater discoloration of 3D-printed resins than of milled composite materials after exposure to staining media (Hashemzade et al. 2025; Ellakany et al. 2023; Shin et al. 2020). The better color stability of CAD/CAM composites can be explained by the manufacturing process. They are industrially fabricated under high temperature and high pressure and exhibit a high degree of polymerization compared to printed polymers (Ruse and Sadoun 2014; Kessler et al. 2025; Shin and Rawls 2009). In addition, 3D-printed resins exhibit greater surface roughness and porosities due to the printing process compared to CAD/CAM composites, which negatively affects color stability (Hashemzade et al. 2025). Furthermore, *Osiceanu et al.* showed that water absorption of composites has an influence on susceptibility to discoloration (Osiceanu et al. 2025). Generally, CAD/CAM composites exhibit lower water absorption, which may be attributed to their denser polymer matrix (Shin et al. 2020; Song et al. 2020; Kessler et al. 2025; Dimitrova et al. 2023).

### Influence of surface treatment

The second hypothesis was confirmed for all PCR groups: untreated specimens consistently exhibited higher ΔE values than polished and glazed specimens. This was also observed in the 0.5-mm GB groups, where untreated specimens generally exhibited higher ΔE values than surface-treated specimens, although not at every time point. By contrast, this pattern was not consistently observed in the 1.0 mm GB groups, where significant differences were limited to day 3 between untreated and glazed specimens, and to day 28 between untreated and polished specimens. The effects of surface treatment were therefore most pronounced in PCR, whereas the effect was less consistent in GB, particularly in the 1.0-mm groups. The positive effect of surface treatment can be explained by the reduction in surface roughness, which limits the adhesion and penetration of color pigments from the dye. When interpreting these findings, it should be taken into account that, in the present experimental setup, only the outer surface of the specimens was polished or glazed, whereas the opposite side remained untreated in order to simulate the internal surface of a restoration. Comparable positive effects of surface treatments in the form of polishing and glazing on color stability have also been described in previous studies (Raszewski et al. 2023; Hashemzade et al. 2025; Lask et al. 2025). This observation underscores the importance of material-adapted post-processing protocols for additively manufactured restorations. The findings in the glazed GB subgroups should be interpreted with caution. In the glazed 0.5-mm GB group, partial loss of the glaze layer occurred during storage, resulting in the loss of two specimens for later measurement time points. Since glazing is not a manufacturer-recommended standard surface treatment for Grandio blocs, the application of Easy Glaze to GB should be regarded as an experimental condition. It cannot be excluded that the interaction between the glaze material and the CAD/CAM composite substrate contributed to chipping of the glaze layer and thereby altered the discoloration behavior of this subgroup. Consequently, the results of the glazed GB groups should not be interpreted as evidence for a clinically recommended glazing protocol for GB, but rather as exploratory data under the present in vitro conditions.

### Influence of layer thickness

The third hypothesis was confirmed for all PCR groups, as well as for the untreated and glazed GB groups. In these groups, specimens with a layer thickness of 0.5 mm showed significantly higher ΔE values than those with a layer thickness of 1.0 mm at all time points after baseline. However, this pattern was not consistently observed in the polished GB groups, where no significant differences were found between the two layer thicknesses on days 1, 2 and 28. Due to the lower material mass, thinner layers allow increased light transmission and may render colored molecules more pronounced. The findings of the present study are supported by the work of *Lee et al.*., who also showed that thinner layers of 3D-printed resins exhibit greater color changes compared with thicker layers. Furthermore, it is conceivable that, at lower layer thicknesses, the diffusion of staining substances becomes relatively more influential (Lee et al. 2022). From a clinical perspective, this finding is relevant because reduced material thickness usually occurs in the margins of restorations. This can make discoloration particularly noticeable and increase susceptibility to staining, especially in older restorations with partial cement loss.

### Limitations

The study is limited by its in vitro design, which must be considered when interpreting the color changes. Biofilm formation, pH fluctuations, clinical surface changes, the cleansing effect of saliva, the tongue, and the lips, as well as individual oral hygiene practices, were not accounted for. This explains why discoloration was observed in all groups during storage, with ΔE*ab values above both the 50:50 acceptability threshold of 2.7 (Paravina et al. 2015) and the commonly used threshold of 3.7 (Khashayar et al. 2014). The increase observed over 28 days should therefore be interpreted as susceptibility to staining under standardized, accelerated coffee exposure, rather than as a direct estimate of clinical staining.

A further limitation is the use of the CIELAB color difference formula (ΔE*ab). Although ΔE*ab remains frequently used in studies on the color stability of dental materials and facilitates comparison with previous work, the CIEDE2000 formula (ΔE00) provides a more perceptually uniform assessment of color differences, particularly in the tooth-colored range. Therefore, the present findings should primarily be interpreted as comparative in vitro color changes between the investigated groups rather than as a direct estimate of visually perceived color differences under clinical conditions.

A methodological aspect of the present study is the use of non-immersed reference areas on the same specimen. Because both materials exhibit a certain degree of translucency, lateral light scattering within the specimen cannot be completely excluded in principle. However, the reference areas were kept constant throughout all measurement sessions, remained outside the staining solution, and showed no measurable color changes over time. This suggests that a potential influence of the stained region on the reference measurements was negligible under the present experimental conditions. Nevertheless, this aspect should be considered when interpreting ΔE values, particularly in thin and translucent specimens.

In addition, only coffee was investigated as a staining medium. Patient-specific nutritional habits regarding the consumption of other staining foods were also not considered. Thermocycling and simulated toothbrushing were not performed. Both factors may alter the surface properties and staining behavior of resin-based materials. Furthermore, Floriani et al. reported reduced shade-matching accuracy using an intraoral scanner and a spectrophotometer after artificial aging, particularly for the darker shade (Floriani et al. 2024). The specimens in the presented study did not have any areas prone to discoloration because of their flat design. In clinical situations color changes are mostly observed in indentations or dimples, such as occlusal grooves or interdental regions.

Future studies should therefore include thermocycling, simulated toothbrushing, additional staining media, and in vivo conditions to better account for clinical influencing factors and to distinguish between extrinsic and intrinsic discoloration.

## Conclusion

The present study showed that, under the tested in vitro conditions, the CAD/CAM nanohybrid composite was more color stable than the additively manufactured resin. Polishing and glazing reduced discoloration, although the effect was less pronounced and less consistent in the CAD/CAM material. Thinner specimens were more susceptible to discoloration. Appropriate surface finishing and sufficient restoration thickness may therefore support color stability, particularly in additively manufactured resin restorations.

## Supplementary Information

Below is the link to the electronic supplementary material.


Supplementary Material 1


## Data Availability

The datasets used and/or analyzed during the current study are available from the corresponding author on reasonable request.

## Abbreviations

ANOVA: Analysis of Variance
CAD/CAM: Computer Aided Design / Computer Aided Manufacturing
GB: Grandio blocs
PCR: Permanent Crown Resin
SPSS: Statistical Package for the Social Sciences
